# Effect of low temperature in the development cycle of *Lucilia sericata* (Meigen) (Diptera, Calliphoridae): implications for the minimum postmortem interval estimation

**DOI:** 10.1080/20961790.2017.1406839

**Published:** 2017-12-21

**Authors:** Laetitia Cervantès, Laurent Dourel, Emmanuel Gaudry, Thierry Pasquerault, Benoît Vincent

**Affiliations:** Forensic Fauna Flora Department, Institut de Recherche Criminelle De La Gendarmerie nationale, Cergy Pontoise Cedex, France

**Keywords:** Forensic science, forensic entomology, accumulated degree days (ADD) method, minimum PMI, low temperature

## Abstract

Knowledge of necrophagous insects’ developmental data is necessary for the forensic entomologist to estimate a reliable minimum postmortem interval (PMI_min_). Among the most represented necrophagous species, *Lucilia sericata* (Diptera, Calliphoridae) is particularly interesting. It is regularly identified in samples, with a predominance in summer, and is commonly used by analysts of our entomology department (Institut de Recherche Criminelle de la Gendarmerie Nationale) to estimate the PMI_min_ with the accumulated degree days (ADD) method. This method requires the mathematical lower thermal threshold to be known. This value dictates the quality of the applied ADD method but cannot be considered as fixed, especially when insect development occurs at temperatures close to the biological threshold. In such conditions, it is necessary to study the influence of such temperatures on development rate, as well as the consequences of estimating the period of first oviposition on cadavers, when using the ADD method. Seven replicate rearings were conducted at six different temperatures: 30 °C, 24 °C, 18 °C, 15 °C, 12 °C and 10 °C. Time of development and time of emergence were recorded. The effect of low temperature on the development cycle and the reliability of the ADD method under this entire temperature spectrum were studied using different linear regression models. Calculated durations of total insect time development and experimental rearing duration were then compared. A global linear model cannot be used on the whole temperature spectrum experienced by *L. sericata* without resulting in an overestimation at some temperatures. We found a combination of two linear regression models to be suitable for the estimation of the total development time, depending on the temperature experienced by *L. sericata*. This approach allowed us to obtain a variation lower than 2% at 12 °C and 10 °C between the calculated duration and experimental duration of development. In comparison, the results obtained with a global model show a variation higher than 3% at 12 °C and 10% at 10 °C.

## Introduction

Estimation of the postmortem interval (PMI) is of crucial importance in criminal investigations. When the limits of traditional legal medicine are reached for decomposing cadavers colonized by insects, forensic entomology can help to estimate the time elapsed since death, the so-called minimum postmortem interval (PMI_min_) [1,2]. The identification of necrophagous fauna collected on the cadaver and its surroundings, together with analysis of reliable environmental data from the location of body [3–5], enables the entomologist to determine the age of the immature insects and, consequently, estimation of the PMI_min_. Therefore, it is essential for forensic entomologists to know the rate of development of the different necrophagous species (Diptera). Being poikilothermic, insect development is affected by ambient temperature [6]. The relationship between development rate and temperature is curvilinear at low and high temperatures and linear in between [7].

Marchenko [8] reported that the development of insects could be described using temperature summation, i.e. the accumulated degree days (ADD) model. To determine an estimate of the date of oviposition, the ADD model is mainly used by our forensic laboratory. The total ADD needed for insect development at one constant rearing temperature (ADD*_i_*) is calculated from the equation, ADD*_i_* = *n(Ti-Ts)*, with *n* being the number of development days, *Ti* the rearing temperature and *Ts* the lower development threshold. This estimation of oviposition period is expressed as a time interval.

One of the insects that colonize a corpse is *Lucilia sericata* (Calliphoridae, Diptera) [3,4]. Numerous published development data for this necrophagous species highlight a large variability in development time [8–14]. This variability could be a result of many factors such as experimental design, geographical strain origin, sampling method and statistical analysis of the data-set [15–20], yet there are few data available for development at temperatures below 15 °C [8].

To use the ADD model, the mathematical minimum threshold for a species has to be determined. This is obtained from a linear regression produced by experimental rearings. An inaccurate minimum threshold could mean an over- or under-estimation of the PMI_min_. This becomes more important when the temperature reaches the mathematical minimum threshold. It is therefore necessary to reconsider the minimum temperature component of the relationship for *L. sericata* and to study it at low average temperatures.

In this paper, we present an assessment of the thermal constants for a French strain of *L. sericata* by a linear approach. Development rates were calculated at two temperatures below 15 °C, and the physiological response was studied. The main aim was to determine if the physiological lower development threshold is similar to the mathematical lower development threshold for our population. We then conducted a validation test of PMI_min_ estimates using different linear models for developmental rates of *L. sericata* to highlight the limitation of a global ADD method and the importance of a geographically specific constant of development.

## Materials and methods

### Strain, conditions of adult rearing and oviposition

Adults of *L. sericata* were captured in Rosny sous Bois (Ile-de-France, region of France) with a bait made of pieces of fish and a hand net. Other larval stage specimens were bought in a fishing tackle shop with the strain coming from Allier, in central France. The latter specimens were used when it became impossible to capture *L. sericata* in its natural environment. This last strain was always mixed with the wild one to try to reduce genetic inbreeding. Adults were kept in plexiglass rearing boxes (300 mm × 300 mm × 500 mm) in an air-conditioned room where the temperature fluctuated between 22 °C and 24 °C. The day/night ratio of 16:8 was kept constant with two banks of twin 15 W fluorescent lights placed on each side of the boxes. Water and sugar were provided *ad libidum.* Adults were able to oviposit on the surface of fresh beef muscle. This piece of meat was observed every hour. As soon as flies were observed on the piece of meat, the observation interval was reduced to 30 min. If eggs were laid on the meat, they were removed from the box and clusters of 50 eggs were prepared under a dissecting microscope LEICA MZ16 using a fine brush dipped in distilled water. Each cluster was placed on approximately 100 g of a fresh piece of beef muscle and transferred into a plastic box (260 mm × 130 mm × 80 mm) containing a layer of sand. Seven replicates were made for each temperature tested (30 °C, 24 °C, 18 °C, 15 °C, 12 °C and 10 °C). Fresh meat was added regularly so that food for larvae was always in excess, to avoid competition.

Captured adults and the fishing tackle shop strain were identified using the identification key of Knut Rognes (1991) before being placed in the rearing box [21].

### Rearing parameters, climatic chamber and monitoring

The plastic boxes were placed in a climatic chamber with a constant internal environment (Sanyo MLR-350^©^ or Sanyo Medicool^©^). The constant temperature set points used for the study were 30 °C, 24 °C, 18 °C, 15 °C, 12 °C and 10 °C (Table 1). These temperatures were chosen to cover the development range described in the literature [8–14]. The lowest temperature was chosen because it was close to the lower mathematical development threshold for *L. sericata.*

**Table 1.** Environmental rearing conditions in the climatic chamber.

Climatic chamber	Temperature set point (°C)	Actual recorded temperature (°C)	Photoperiod day/night
Sanyo MLR-350^©^	30	29.6 ± 0.2	16:8
	24	23.9 ± 0.2	16:8
	18	18.0 ± 0.2	11:13
	15	15.0 ± 0.2	11:13
Sanyo Medicool^©^	12	12.2 ± 0.2	9:15
	10	10.0 ± 0.2	8:16

The temperature of the climatic chamber was monitored by thermal probes (error ± 0.2 °C) connected to a LOGOSCREEN NT^©^ temperature station.

The temperatures used in the analysis were the average temperatures recorded by the probes throughout the rearing, until the last adult emergence in the boxes at each temperature. The photoperiod was adapted to the day/night period in France regarding the season and the temperature [22]. Temperature data recorded by a weather station on the site of capture allowed us to establish a monthly average temperature from 2006 to 2008. From these we could estimate the period of the year when the temperature of interest was reached. Using day/night duration data over a year in Paris, France, we also have the photoperiod associated with the time of year [23]. This information is summarised in Table 1.

Humidity inside the climatic chamber was always above 40% and was monitored with a 175-H2 TESTO^©^ data logger.

### Monitoring period and parameters studied

The frequency of observations on the replicates varied according to the temperature. The procedure consisted of visual observations to determine the stage reached (egg hatching, third instar larval, pupal stage and emergence). At 24 °C and 30 °C, the replicates were inspected every two hours, day and night. At 18 °C, they were inspected only twice a day, at 8 am and 6 pm; from 15 °C to 10 °C, once a day at 8 am. When a stage defined above was about to occur, the frequency of inspections was doubled and the boxes were also inspected during the night. The determination of the start of the third larval stage was made by examining the larvae under LEICA MZ16 and MZ12 stereomicroscopes (magnification 8–125 and 8 × magnification 100). This took 5–10 min per replicate. The specimens were kept alive to obtain results on the most complete population. Each larva was inspected and temporarily placed on a fresh piece of meat. This was continued until all larvae had been inspected or a third-stage larva was found. At this point, all larvae were returned to the initial rearing box. Manual handling was as limited as possible.

In each box, egg hatching, first appearance of a third-stage larva (the first specimen), pupal stage and adult emergence were used as reference points for the recording of developmental stages. The first and second larval stages were not studied because they were too fragile to be handled. Puparial stage was considered reached when there was an irreversible stabilization of the entire still white or light brown cuticle [24]. Pupae and successfully emerging adults were recorded daily and removed from the boxes. All these steps were performed at all temperatures.

The following abbreviations were used to indicate the intervals between each development stage (in hours):

**O**-**E**: time elapsed between **O**viposition and **E**gg hatch
**E**-**L3**: time elapsed between **E**gg hatch and third instar (**L3**)
**L3**-**Pu**: time elapsed between **L3** and **Pu**pal stage (postfeeding stage included)
**Pu**-**Em**: time elapsed between **Pu**pal stage and adult **Em**ergence
**O**-**Em**: time elapsed between **O**viposition and adult **Em**ergence (O-Pu + Pu-Em)

### Description of the analytical method

The minimum duration of a complete development cycle was obtained from our experimental rearing at each temperature and in each box (seven replicates per temperature).

The means of these seven values per temperature were fitted in a graph with *y*-axis: 1/development time (*t*) and *x*-axis: temperature (T °C). From the means of our actual data, three linear regressions, *t* = *f* (T °C), were performed (XLSTAT^©^ versus 6.1 of Addinsoft) (Table 2). A linear regression *R*_3__min_ was performed with the three lowest temperatures (15 °C, 12 °C and 10 °C). A linear regression *R*_4_ was performed without the lowest temperatures (30 °C, 24 °C, 18 °C and 15 °C). A linear regression *R*_5_ was performed adding the data obtained at 12 °C and one other, *R*_6_, adding the data obtained at 10 °C to all the other temperatures.

**Table 2.** Development cycle time (in hours) for *Lucilia sericata* at six different temperatures.

	*T* = 30 °C			*T* = 24 °C			*T* = 18 °C			*T* = 15 °C			*T* = 12 °C			*T* = 10 °C		
Items	$\bar{x}$	σ	**%**	$\bar{x}$	σ	%	$\bar{x}$	σ	%	$\bar{x}$	σ	%	$\bar{x}$	σ	%	$\bar{x}$	σ	%
O-E	11.2	0.4	4.2	15.8	0.1	4.8	40.0	6.3	6.1	46.2	0.0	4.9	70.2	0.0	4.7	71.0	0.0	2.4
E-L3	28.8	0.9	10.7	42.3	2.3	12.9	84.1	6.1	12.9	144.0	0.0	15.3	196.3	20.1	13.2	md	md	md
L3-Pu	85.0	11.9	31.7	85.0	2.4	25.9	183.1	36.8	28.1	226.3	12.8	24.1	448.3	69.0	30.1	md	md	md
Pu-Em	143.0	6.5	53.3	185.5	9.5	56.4	345.1	20.1	52.9	521.1	18.1	55.6	773.8	25.0	52.0	865.3	86.0	29.8
O-Em	268.1	10.3	100.0	328.7	9.8	100.0	652.3	30.1	100.0	937.7	25.7	100.0	1 488.6	90.5	100.0	2 904.0	126.0	100.0

$\bar{x}$: average; σ: standard deviation; %: percentage of development for a stage; *T*: temperature; md: missing data

**O**-**E**: time elapsed between **O**viposition and **E**gg hatch; **E**-**L3**: time elapsed between **E**gg hatch and third instar (**L3**); **L3**-**Pu:** time elapsed between **L3** and **Pu**pal stage; **Pu**-**Em:** time elapsed between **Pu**pal stage and adult **Em**ergence; **O**-**Em:** time elapsed between **O**viposition and adult **Em**ergence.

From this, the lower mathematical development threshold (*Ts*) was extrapolated by an *x*-intercept approach for each linear regression. *Ts* is the value of T°C when the rate of development is zero. The total accumulated degree-days needed for a complete development (ADD*_i_*) is the inverse of the line's slope (a), so ADD*_i_* = 1/*a*.

For every thermal constant obtained from the linear regression, dates of oviposition were calculated using the ADD method for every temperature (30 °C, 24 °C, 18 °C, 15 °C, 12 °C and 10 °C).

These calculated development times were then compared with data obtained from experimental rearings for the same temperatures (seven rearing boxes per temperature, so mean development time from the seven boxes was used).

Finally, additional dates of oviposition were calculated using the ADD method with Marchenko's developmental data. The abbreviation ADD*_m_* was used to define the regression obtained by Marchenko for *L. sericata*. Marchenko's data were used as they presented a thermal constant for a temperature spectrum similar to the present study (11 °C–30 °C) [8].

## Results

### Mean ($\bar{x}$), standard deviation (σ) and percentage (%) obtained from the raw data obtained by rearing

The duration of each stage varied with the temperature (Table 2). For 10 °C experiment, to preserve the very low number of larvae in each replicate and obtain a complete cycle of time development, the first and second instars were not handled due to their fragility, resulting in some missing data.

We found that the higher the temperature, the lower the duration of each stage. The egg stage represents 2.5% to 6% of the complete development cycle time, larval stage between 38.5% and 45% (except at 10 °C: 68%), pupal stage between 52% and 56.5% (except at 10 °C: 30%).

The standard deviations depend on the frequency of the observation interval. Thus, a null standard deviation does not mean all specimens have reached the next stage at the same time but that every rearing box presents the stage required during the observation. A high standard deviation indicates the data points are spread out over a wider range of values. This means for one rearing temperature more observations were needed to obtain the required stage for all the boxes.

### Linear regressions and date of oviposition comparisons

For every temperature between 30 °C and 10 °C (*R*_6_ model), the minimum development times of *L. sericata* have a linear relationship with temperature (*R*^2^ = 0.988) (Table 3). In this temperature range, the lower development threshold for total development (*Ts*) is 8.6 °C and the total accumulated degree-days (ADD*_i_*) is 228 d. The *R*_6_ and *R*_3_ _min_ models have the highest *R*^2^ values.

**Table 3.** Information on the linear regression models used for analysis of results.

Regression name	Equation	*R*^2^	*Ts* (°C)	ADD*_i_* (d)
*R*_4_	*y* = −3.99 × 10^−2^ + 4.47 × 10^−3^*x*	0.978	8.9	223
*R*_5_	*y* = −3.845 × 10^−2^ + 4.4 × 10^−3^*x*	0.985	8.7	226
*R*_6_	*y* = −3.7 × 10^−2^ + 4.388 × 10^−3^*x*	0.988	8.6	228
*R*_3__min_	*y* = −3.4 × 10^−2^+ 4.12 × 10^−3^*x*	1	8.3	243
ADD*_m_*	*y* = −4.3 × 10^−2^ + 4.8 × 10^−3^*x*	–	9.0	208

**Figure 1.**
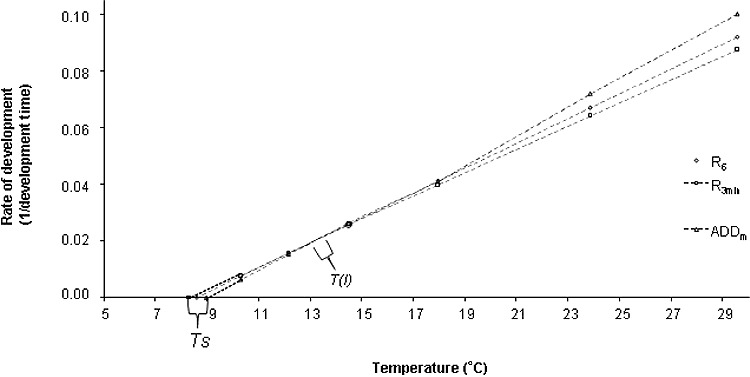
Regression curve for *R*_6_, *R*_3 min_ and ADD*_m_* models and experimental results. *Ts:* lower development threshold of each regression line, *R*_3 min_, *R*_6_ and ADD*_m_*;*T*(*I)*: temperature at the intersection point between two regression lines (*R*_6_/*R*_3 min_ or ADD*_m_*/*R*_3 min_).

The intersection point (I) between *R*_6_ and *R*_3 min_ equals 13.3 °C (when *y* (*R*_6_) = *y* (*R*_3__min_)). The (I) between ADD*_m_* and *R*_3 min_ equals 13.2 °C (Figure 1).

The thermal constants (*Ts* and ADD*_i_*) vary according to the model used. *Ts* is between 8.3 °C and 8.9 °C for all the linear regressions studied. The ADD*_i_* ranges between 223 and 243 d.

The thermal constant obtained with the *R*_4_ model is the closest to the thermal constant obtained with the ADD*_m_* model.

### Comparison between R_4_, R_5_ and R_6_ models and experimental results

Figure 1 shows that including the lowest temperatures studied (10 °C and 12 °C) in the calculation of oviposition date increases the number of temperatures used to create the linear regression, which causes a decrease in the difference between estimated results and experimental results (Table 4). For example, at 10 °C, the difference in development time between estimated results using *R*_4_ (30 °C, 24 °C, 18 °C and 15 °C) and experimental rearings is 39.4 d, whereas using *R*_6_ (30 °C, 24 °C, 18 °C, 15 °C, 12 °C and 10 °C) this difference is only 13.1 d. This trend is the same at 12 °C.

**Table 4.** Duration (in days) of the total development time obtained with each linear regression model and experimental rearing.

Temperature (°C)	*R*_4_ model	*R*_5_ model	*R*_6_ model	*R*_3__min_ model	ADD*_m_* model	Experimental results
30	10.8	10.9	10.9	11.4	10.0	11.2
24	14.9	14.9	15.0	15.6	13.9	13.7
18	24.3	24.5	24.4	25.2	23.1	27.2
15	39.8	39.0	38.7	39.2	37.8	39.0
12	68.6	65.4	64.0	62.9	65.0	62.0
10	160.4	141.3	134.1	121.5	160.0	121.0
S (days)	51.3	27.9	19.7	5.8	48.6	0.0

S (days): Sum of the difference at each temperature between results obtained with models and experimental results in days (in absolute values).

Furthermore, the sum of the absolute values of the differences between experimental results from rearing and estimated results are 56.2 d with *R*_4_, 27.9 d with *R*_5_ and 19.7 d with *R*_6_.

All these models overestimate the development time obtained at 24 °C, 12 °C and 10 °C, and underestimate it at 30 °C and 18 °C.

The results obtained with the *R*_6_ model are closest to the experimental rearing results.

### Comparison of results obtained with R_6_, R_3_ _min_, ADD_m_, models and the experimental data

**Figure 2.**
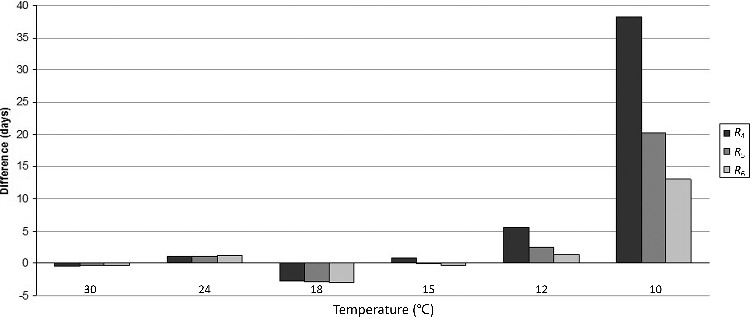
Difference in days of the development time between estimate results using *R*_4_, *R*_5_ and *R*_6_ models and results obtained from experimental rearing.

Results obtained with *R*_3_ _min_ (10 °C, 12 °C and 15 °C) are the closest to the experimental data. The most important difference is obtained with the ADD*_m_* model. The sum of the absolute values of the difference between experiment data and the *R*_3_ _min_ model data is 5.8 d, 48.6 d with data obtained with ADD*_m_* (Figure 2).

Only the *R*_3_ _min_ model gives a similar duration of the total development time with the experimental result at 10 °C (difference of 0.41%).

For the temperatures studied, the results obtained with this model (*R*_3_ _min_) showed variation from the experimental data of 0.41% to 14% (absolute value): this means a variation of less than 2 d at each temperature compared to the experimental results. At 30 °C, 15 °C, 12 °C and 10 °C, this difference was less than one day.

The *R*_6_ model varied from experimental data by between 0.9% and 10.8% for the temperatures studied, with the greatest difference at 10 °C (13.1 d) and the smallest at 30 °C and 15 °C (less than 1 d).

The ADD*_m_* model is accompanied by a variation of between 1.5% and 10.5% from 30 °C to 12 °C. This one exceeds 30% at 10 °C, which represents 39 d for a cycle of 121 d.

At 24 °C, 12 °C and 10 °C all the models overestimate development time, whereas at 30 °C and 18 °C all the models underestimate development time. At 15 °C, no trend is perceptible.

The sum of the absolute values of the difference between experimental results from rearing and estimate results are 4.1 d with ADD*_m_*, 4.3 d with *R*_6_ and 4.2 d with *R*_3_ _min_.

## Discussion

### Effect of low temperatures on the development cycle

These results confirm the existence of a linear relationship between the temperature and the development time. An increase in temperature causes an increase in metabolic activity and reduces the development time [12]. In terms of duration, the pupal stage is predominant up to as low as 12 °C (>50% of the total development of the insect). These data are consistent with other published data [9,10,12,25]. However, at 10 °C, contrary to these standard references, the pupal stage represents only 30% of the total development of the insect, and the larval stage is lengthened. A rearing temperature close to the mathematical minimum threshold could be an explanation for this. Furthermore, too little data are available at this temperature because of few specimens survived to emergence. So, at 10 °C, results need to be confirmed regarding the low number of insect with a complete development cycle (only 9/350 emerged at all).

Complete cycles of development were also obtained at 15 °C (39 d of development), 12 °C (62 d) and 10 °C (121 d). These results are not generally found in the literature. Only Marchenko [6] presented a development time to emergence for both 11 °C (103.5 d) and 12 °C (69 d). But when Marchenko's data are plotted in a linear regression, the *R*² value is 1. Therefore, it is possible that these data were calculated rather than obtained experimentally.

Anderson [12] found that at 15.8 °C several specimens under laboratory conditions enter diapause in the prepupal stage. Niederegger [26] showed that at 13.0 °C there is no pupation for *L. sericata*. Grassberger and Reiter [9] recorded no emergence of adults at temperatures below 15 °C. In accordance with these studies, we observed during rearing that several specimens did not progress after the third-instar larvae at 15 °C and 12 °C (not enough data at 10 °C). This phenomenon also occurred at 18 °C, and could be explained by short days (11:13 to 8:16) and low temperature effects (12 °C–18 °C) [27,28]. In *L. sericata*, short days acting directly on larvae have been shown to be important in diapause induction [28].

A temperature of 10 °C impedes the hatching of larvae from eggs, but 9 specimens out of 350 completed their development cycle with a lengthened larval stage. This result highlights that a low temperature (10 °C) is close to the lower physiological development threshold.

Nevertheless, these results are obtained at constant temperatures. Niederegger [26] found that rearing at fluctuating temperatures between 5 °C and 29 °C showed no development of *L. sericata* because the periods of 5 °C in a climatic chamber impeded the hatching of larvae from eggs. For mean temperatures equal to or higher than 15 °C, Davies and Ratcliff [29] showed an increase of the larval growth rate at fluctuating temperatures, a stark contrast to Greenberg [10,24]. The latter indicated that fluctuating temperatures tend to delay larval growth in four species, with a significant effect on *L. sericata*.

### Thermal constant

This study confirms that the theoretical thermal constant values are not fixed but depend on the model that is applied [30,31].

The model built with four temperatures, *R*_4_ (30 °C, 24 °C, 18 °C and 15 °C), allows us to obtain a total accumulated degree-days (ADD*_i_*) of 223 d and a lower development threshold (*Ts*) of 8.9 °C. The model with five temperatures, *R*_5_ (30 °C, 24 °C, 18 °C, 15 °C and 12 °C), gives an ADD*_i_* of 226 d and a *Ts* of 8.7 °C, and the model that includes 10 °C, *R*_6_, gives values that were very similar to *R*_5_ (228 d and 8.6 °C). This study indicates that the lower the extreme temperature, the lower the *Ts* and the higher the ADD*_i_*. These values are very different from the model *R*_3_ _min_ (243 d and 8.3 °C). What we highlight here is the effect of the number of temperatures used in the building of a model and the calculation of the thermal constant. Richards et al. [30,31] showed that a correct evaluation of the thermal constant and thermal threshold temperature depends on the number and range of temperatures used and the time intervals between each observation.

In this study, the frequency of observation varied according to the temperature. Our null standard deviations between 15 °C and 10 °C for the egg stage show that we should have reduced the monitoring time between oviposition to egg hatch at low temperatures.

Our result showed a *Ts* between 8.3 °C and 8.9 °C for ADD*_i_* between 223 and 243 d. These thermal constants are in the same range as those obtained with development data by Grassberger (ADD*_i_*: 214 d; *Ts*: 9.14 °C) [9] and Gosselin et al. [17] (ADD*_i_*: 217.97 d; *Ts*: 9.55 °C) but are very different from the thermal constant found by Anderson [12] (ADD*_i_*: 352.97 d; *Ts*: 4.48 °C) and Greenberg [10] (ADD*_i_*: 485 d; *Ts*: 11.3 °C). The conditions of rearing, photoperiod and examination interval can explain these differences. Indeed, the developmental plasticity of *L. sericata* seems to depend on genetic variations between populations, but variation is also caused by environmental conditions, rearing conditions and the statistical analysis approach employed [15–19].

### Choosing a model to use in the estimation of the date of oviposition

To estimate the date of oviposition, a forensic entomologist needs a replicable model with the lowest possible uncertainty, that underestimates rather overestimates the timing (it is not acceptable to obtain an egg laying date that is calculated prior to the death, excepting cases where myiasis occurred prior death).

The two closest models to our experimental data are *R*_3_ _min_ and *R*_6_. For the *R*_3_ _min_ model, there is a difference between 0.4% and 14% in comparison with our experimental results in the calculation of the insect development time (e.g. 0.5 d at 10 °C). For the *R*_6_ model, this difference is between 0.9% and 10.8% (e.g. 13.1 d at 10 °C).

Thus, the *R*_3_ _min_ is the most suitable model covering the whole temperature range, although a 14% deviation at 24 °C remains important (it represents an error of about two additional days for a development cycle that lasts 15 d).

However, no model provides a development time that matches or underestimates the total insect development time when compared with the experimental data, within the range of temperatures we tested.

Between 30 °C and 12 °C, the duration of the total development cycle calculated with the ADD*_m_* model showed differences of 1.46%–10.47% in comparison with experimental data. This is similar to the values obtained with the *R*_6_ model (0.90%–10.83%).

In this study, the variation exceeds Marchenko's data at 30 °C, 18 °C and 10 °C [8]. Between 30 °C and 15 °C, the results with ADD*_m_* underestimate or match the experimental duration of the total development cycle. The *R*_6_ model overestimates the duration of development at 24 °C. Thus, the ADD*_m_* model is better than the *R*_6_ model from 30 °C to 15 °C to estimate a PMI_min_ with the lowest possible uncertainty.

Below 15 °C, the two models (*R*_6_, ADD*_m_*) overestimate the duration of the development cycle (more than two days on a total insect development time). At 10 °C, the ADD*_m_* artificially prolongs the duration of the development cycle by more than one month in a cycle of four months, while *R*_6_ prolongs this cycle by 13 d only.

Thus, at low temperatures, the global linear model *R*_6_ and ADD*_m_* are less adapted in comparison to our experimental data than the linear model built with the three lowest temperatures (*R*_3_ _min_).

**Figure 3.**
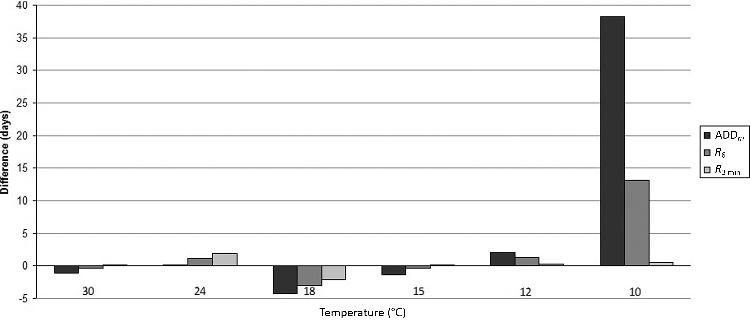
Difference in days between the duration of a total development cycle obtained with *R*_3 min_, *R*_6_, ADD*_m_* models and experimental results at different temperatures.

In this study, *R*_3_ _min_ overestimates the result: the sum of the absolute values of the difference between this model and the experimental results is 4.19 d. This number decreases to 4.05 with the ADD*_m_* model. Consequently, above *T(I)* (Figure 1), the ADD*_m_* model gives the closest results to the experimental data. Below *T(I)* (Figure 3), the ADD*_m_* model prolongs the cycle development artificially, with an overestimation of the development cycle duration. The *R*_3_ _min_ model is more reliable with a lower overestimation.

Regarding the data obtained at 18 °C, the developmental time is underestimated by all models, with an emergence rate lower (51%) in comparison with the ones obtained at 15 °C and 24 °C (±75%). Nevertheless, this discrepancy is not unusual considering the number of replicates (seven rearing boxes) and the replication of the same protocol in the same conditions for each rearing temperature. Only another similar experiment could confirm or refute this result.

Thus, the combined use of two different models (ADD*_m_* and *R*_3 min_) seems to be the most suitable for experimental data, rather than using only one model to cover a wide temperature range.

Nevertheless, this conclusion is still confined to the limits of the temperature/rate model and cannot be considered as a general rule for all development models. Other models may propose an alternative [32,33] for the insect age calculation in non-linear parts of the temperature-dependent development. This approach could be an alternative method to estimate the relationship between temperature and time on the development of necrophagous species. But it requires rearing at the extreme limits of the temperature range of the species, while taking into account realistic geographical parameters, especially in warmer and colder areas. Furthermore, parameters like photoperiod [22] and rearing substrate tissue type [34] could affect the insect development duration. Our evaluation is promising, but the true impact needs further investigation.
